# LncRNA PCED1B-AS1 mediates miR-3681-3p/MAP2K7 axis to promote metastasis, invasion and EMT in gastric cancer

**DOI:** 10.1186/s13062-024-00468-z

**Published:** 2024-05-02

**Authors:** Jia Cao, Yicheng Yang, Bensong Duan, Haibin Zhang, Qinwei Xu, Junyi Han, Bing Lu

**Affiliations:** 1 Endoscopy Center, Department of Gastroenterology, Shanghai East Hospital, School of Medicine, Tongji University, Shanghai, China; 2 Department of Gastrointestinal Surgery, Shanghai East Hospital, School of Medicine, Tongji University, Shanghai, China; 3 Department of General Surgery, Shanghai East Hospital, School of Medicine, Tongji University, Shanghai, 200120 China

**Keywords:** Gastric cancer, lncRNA PCED1B-AS1, miR-3681-3p, MAP2K7

## Abstract

**Background:**

LncRNA PCED1B-AS1 is abnormally expressed in multiple cancers and has been confirmed as an oncogene. Our study aimed to investigate the regulatory mechanism of lncRNA PCED1B-AS1 in gastric cancer.

**Methods:**

TCGA database was used to analyze the abnormal expression of lncRNA PCED1B-AS1 in gastric cancer. By database prediction and mass spectrometric analysis, miR-3681-3p and MAP2K7 are potential downstream target molecules of lncRNA PCED1B-AS1 and verified by dual-luciferase report assay. RT-*q*PCR analysis and western blot were performed to detect the expressions of PCED1B-AS1 and MAP2K7 in gastric cancer cell lines and tissues. CCK-8 kit was applied to measure the cell viability. Wound healing and Transwell experiment were used to detect the migration and invasion. Western blot and immunohistochemical staining were performed to detect the expressions of EMT-related proteins in tissues. The changes of tumor proliferation were detected by xenograft experiment in nude mice.

**Results:**

PCED1B-AS1 expression was higher but miR-3681-3 expression was lower in gastric cancer cell lines or tissues, compared to normal group. Function analysis verified PCED1B-AS1 promoted cell proliferation and inhibited cell apoptosis in gastric cancer cells in vitro and in vivo. LncRNA PCED1B-AS1 could bind directly to miR-3681-3p, and MAP2K7 was found to be a downstream target of miR-3681-3p. MiR-3681-3p mimics or si-MAP2K7 could partly reverse the effect of PCED1B-AS1 on gastric cancer cells.

**Conclusion:**

PCED1B-AS1 accelerated cell proliferation and inhibited cell apoptosis through sponging miR-3681-3p to upregulate MAP2K7 expression in gastric cancer, which indicated PCED1B-AS1/miR-3681-3p/MAP2K7 axis may serve as a potential therapeutic target for gastric cancer.

**Supplementary Information:**

The online version contains supplementary material available at 10.1186/s13062-024-00468-z.

## Introduction

Gastric cancer is one of the most common malignancies in the world and the third leading cause of cancer-related death [1]. With the rapid development of medical technology, immunotherapy [2] and targeted therapy [3] have gradually been widely used in the treatment of gastric cancer. However, approximately 40% of gastric cancer patients still have metastasis, and the 5-year survival is not ideal [4]. Considering the malignant effects of gastric cancer, timely diagnosis and effective treatment is the key to improve the survival of patients. Therefore, elucidating the complex pathological mechanism of gastric cancer and seeking specific biomarkers may provide new ideas for early diagnosis and development of new drug targets. It is urgent to further explore the molecular mechanism of gastric cancer.

Long noncoding RNAs (lncRNAs) are a class of RNA transcripts that are longer than 200 bp in length and have no protein-coding ability [5]. A large number of evidences have shown that lncRNA could regulate cell biological processes in a variety of complex diseases, including proliferation, autophagy and apoptosis, and affect pathological progress. Especially in cancer-related studies, reports have confirmed that lncRNA may be involved in malignant tumor invasion, metastasis, and even drug resistance. For instance, lncRNA HAR1A mediates the expression of ALK1 to regulate EMT process and participate in the development of oral cancer [6]. Zou et al. [7] found that lncRNA HOTTIP promoted the proliferation and invasion of ovarian cancer cells and could be acted as a key prognostic marker in ovarian cancer. Long non-coding RNA LINC00982 promoted the high expression of CTSF through transcription factor HEY1 and prevents the malignant progression of gastric cancer [8]. It has been reported [9] that lncRNA UBE2CP3 plays crucial a role in GC progression by modulating miR-138-5p/ITGA2 axis. Li et al. [10] found that lncRNA MAGI2-AS3 promoted tumor progression through sponging miR-141/200a and maintaining overexpression of ZEB1 in gastric cancer. Moreover, lncRNA NR2F1-AS1 promotes GC progression through miR-29a-3p/VAMP7 axis, which may be related to EMT progression in gastric cancer [11]. Liu et al. [12] reported that knockdown of PCED1B-AS1 regulated HOXA9 through sponging miR-633 and inhibited the progression of colorectal adenocarcinoma. However, the molecular mechanism of PCED1B-AS1 regulation in gastric cancer is rarely reported.

Combined with database prediction, RT-*q*PCR results confirmed the abnormally high expression of lncRNA PCED1B-AS1 in gastric cancer, suggesting that PCED1B-AS1 plays a critical role in gastric cancer. Our study intended to investigate the molecular mechanism of PCED1B-AS1 expression specificity in gastric cancer, invasion, proliferation and EMT progression of gastric cancer cells. Our study found that PCED1B-AS1 negatively regulates miR-3681-3p, promotes the expression of MAP2K7, and exacerbates the malignant progression of gastric cancer.

## Materials and methods

### The Cancer Genome Atlas database analysis

The TIMER2 and GEPIA2 websites were used to observe the expression difference of PCED1B-AS1 and MAP2K7 between tumor and adjacent normal tissues of The Cancer Genome Atlas (TCGA) data and the Genotype-Tissue Expression (GTEx) data.

### Tissue samples

Tumor samples and adjacent adjacent tissues from 10 gastric cancer patients who underwent surgical resection in Shanghai East Hospital from August 2019 to December 2020 were collected. Three pathologists evaluated tumor tissue and adjacent normal tissue. All specimens were snap-frozen in liquid nitrogen and stored at − 80 °C for further use. All patients signed informed consent. The clinical study was approved by the Ethics Committee of Shanghai East Hospital (No. 2020-173).

### Cell culture and cell transfection

Human gastric cancer cell lines (AGS, HGC-27, SGC-7901 and MKN45) and Normal gastric mucosal epithelial cell (GES-1) were obtained from ATCC (USA). The cells were cultured in DMEM with 10% FBS in an incubator at 37 °C with 5% CO_2_. The plasmids were synthesized and supplied by Sangon Biotech and transfected into cells using Lipofectamine 3000 as the manufacturer's instructions.

### CCK-8 kit

After transfection, cell viability was determined by CCK-8 kit. In brief, AGS or HGC-27 cells were cultured in 96 plates and incubated in incubators at 37 °C. And 10 μl CCK-8 was added and cultured for 2 h. The absorbance at 450 nm was measured with a microplate reader to evaluate the cell activity.

### Mass spectrometric analysis

The protein samples were passed through an Orbitrap Fusion™Lumos™Tribrid™ mass spectrometer (Thermo Fisher Scientific) with an Easy-NanOLc 1200 system (Thermo Fisher Scientific) online LC–MS/MS analysis of nanosprays. The samples were loaded with 2 μL peptide (analytical column: Acclaim PepMap C18, 75 μm × 25 cm) and separated by gradient for 60 min. The column temperature was 40 °C and the flow rate was maintained at 300 nL/min. Use an electrospray voltage of 2 kv on the inlet of the mass spectrometer.

### RNA fluorescence in situ hybridization (FISH)

In order to determine the localization of PCED1B-AS1 in cells, fluorescence in situ hybridization kit was used for detection. Briefly, FAM labeled PCED1B-AS1 probe signal (green) was detected according to the kit manufacturer's instructions. The nuclei were stained with DAPI (blue). Confocal microscopy (Leica) was used to observe and photograph.

### Dual-luciferase reporter assay

Based on bioinformatics database predictions, we synthesized WT and mutant sequences containing miR-3681-3p binding and transfected their cloned plasmids into AGS and HGC-27 cells. Around 48 h after transfection, relative luciferase activity was determined by dual luciferase reporter gene assay system.

### Wound healing assay

After transfection, the AGS or HGC-27 cells (2 × 10^5^ cells/mL) were cultured on 24-well plates and incubated in an incubator at °C. Cell scratch model was established with 10 μL pipette tip. Serum-free medium was added and cultured for 24 h and 48 h. Cells were observed and photographed under a microscope (Leica).

### Transwell experiment

Transwell experiment was used to detect invasion of AGS or HGC-27 cells. Cells were placed in the Transwell chamber coated with Matrigel at a density of 5 × 10^4^ cells/well. Then, 200 μL serum-free medium was added to the top layer and 400 μL medium containing 10% FBS was given to the bottom layer for culture. Twenty hours later, fixed with 95% ethanol and stained with crystal violet. The cells were randomly observed and counted under an inverted microscope (Leica).

### Western blotting assay

Proteins were extracted from cells using RIPA lysates and then measured using BCA kits. The proteins were separated by SDS-PAGE and then transferred to PVDF membrane. 5% skim milk was sealed for 3 h and then co-incubated with specific primary antibodies overnight. After washing the membrane with TBST, the secondary antibody was incubated at room temperature for 1 h. Enhanced chemiluminescence was used to visualize the membrane, and Image J software was used to measure the gray value to evaluate the relative expression.

### RT-*q*PCR analysis

As previously reported, we used TRIzol to extract RNA from cells. cDNA was synthesized from total RNA using the Takara PrimeScript RT reagent kit. Takara SYBR Green PCR kit was used for real-time quantitative PCR. The 2^−ΔΔCt^ method was used to calculate gene expression levels. GAPDH or U6 was used as the internal control.

### Immumohistochemical staining

In short, the tumor tissues of mice were sequentially treated with the following steps: fixation with 4% paraformaldehyde, paraffin embedding, sectioning, dewaxing, antigen repair with 3% H_2_O_2_, incubation with primary antibody at 4 °C overnight, incubation with secondary antibody, hematoxylin staining, and neutral adhesive sealing. Finally, the stained samples were observed, photographed and analyzed by professional pathological researchers under the light microscope.

### Xenograft tumor model

Six-week-old male nude mice were purchased from Beijing Huafukang Experimental Animal Science and Technology Co., LTD., and randomly divided into si-NC and si-PCED1B-AS1 groups. The transfected cells were subcutaneously injected into mice according to groups. Tumor changes were measured and recorded every 6 days. The mice were euthanized after 30 days. The tumor tissue was isolated and the volume and weight were measured. The animal experiments were approved by the Animal Ethics Committee of Shanghai East Hospital (No. 20201229).

### Statistical analysis

The data in the study were expressed as $\overline{x}$ ± SEM. SPSS22.0 software package was used for statistical analysis. The difference between the two groups was analyzed by unpaired *t* test. Multiple comparisons were calculated using one-way analysis of variance (ANOVA), followed by Tukey's post hoc test. *P* < 0.05 indicated statistical difference.

## Results

### PCED1B-AS1 was overexpressed in gastric cancer tissues

**Fig. 1:**
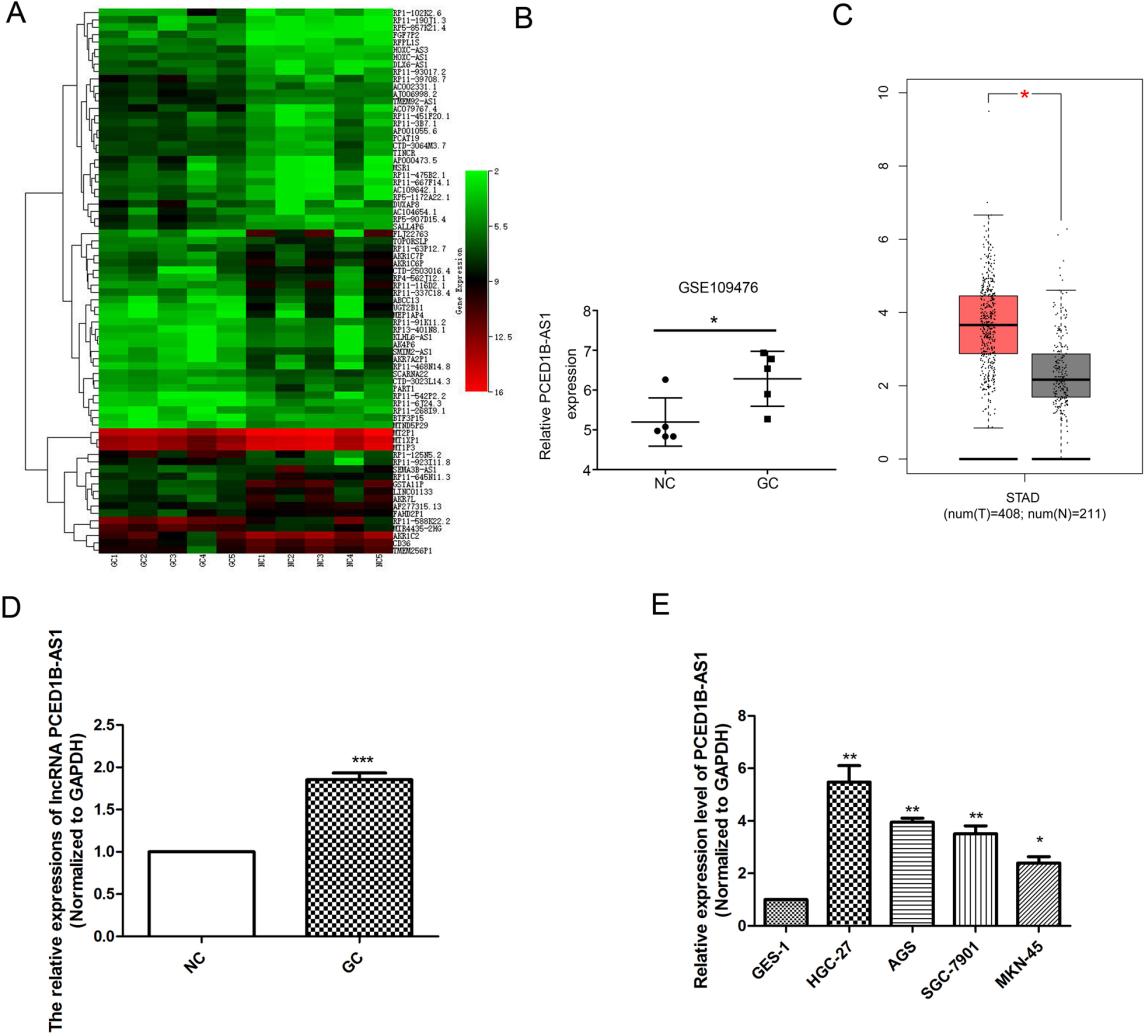
PCED1B-AS1 was overexpressed in gastric cancer tissues. **A** Heat map showed lncRNA expression levels in tumor tissue and its paired normal tissue. **B** Genotype-Tissue Expression (GTEx) data predicted the mRNA expression of PCED1B-AS1 in tumor tissue and its paired normal tissue (n = 5). **C** TCGA database predicted the expression level of PCED1B-AS1 in tumor tissues (n = 408) and normal tissues (n = 211). **D** RT-qPCR analysis was used to detect the expressions of lncRNA PCED1B-AS1 in the tissues of patients (n = 10). **E** RT-qPCR analysis was used to detect the levels of PCED1B-AS1 in the gastric cancer cells. The data were presented as mean ± SEM. versus NC/GES-1 group, *P < 0.05, **P < 0.01, ***P < 0.001

Firstly, through screening gene expression microarrays related to GC from the GEO database, the dataset (GSE109476) was ultimately selected. As presented in Fig. 1A, we selected PCED1B-AS1 as a differentially differentiated gene in tumor tissues. In addition, database prediction (Fig. 1B, C) showed that lncRNA PCED1B-AS1 was significantly up-regulated in gastric cancer. Furthermore, the expressions of PCED1B-AS1 in gastric cancer tissues was detected by RT-*q*PCR, and the results (Fig. 1D) were consistent with the database prediction. Besides, RT-qPCR results (Fig. 1E) showed that compared with GES-1 group, the level of PCED1B-AS1 in gastric cell line was increased significantly, especially in AGS and HGC-27 cells. Taken together, it suggested that PCED1B-AS1 was overexpressed in gastric cancer tissues and cell lines.

### Knockdown PCED1B-AS1 inhibited cell viability, invasion and migration in gastric cancer cell line

**Fig. 2:**
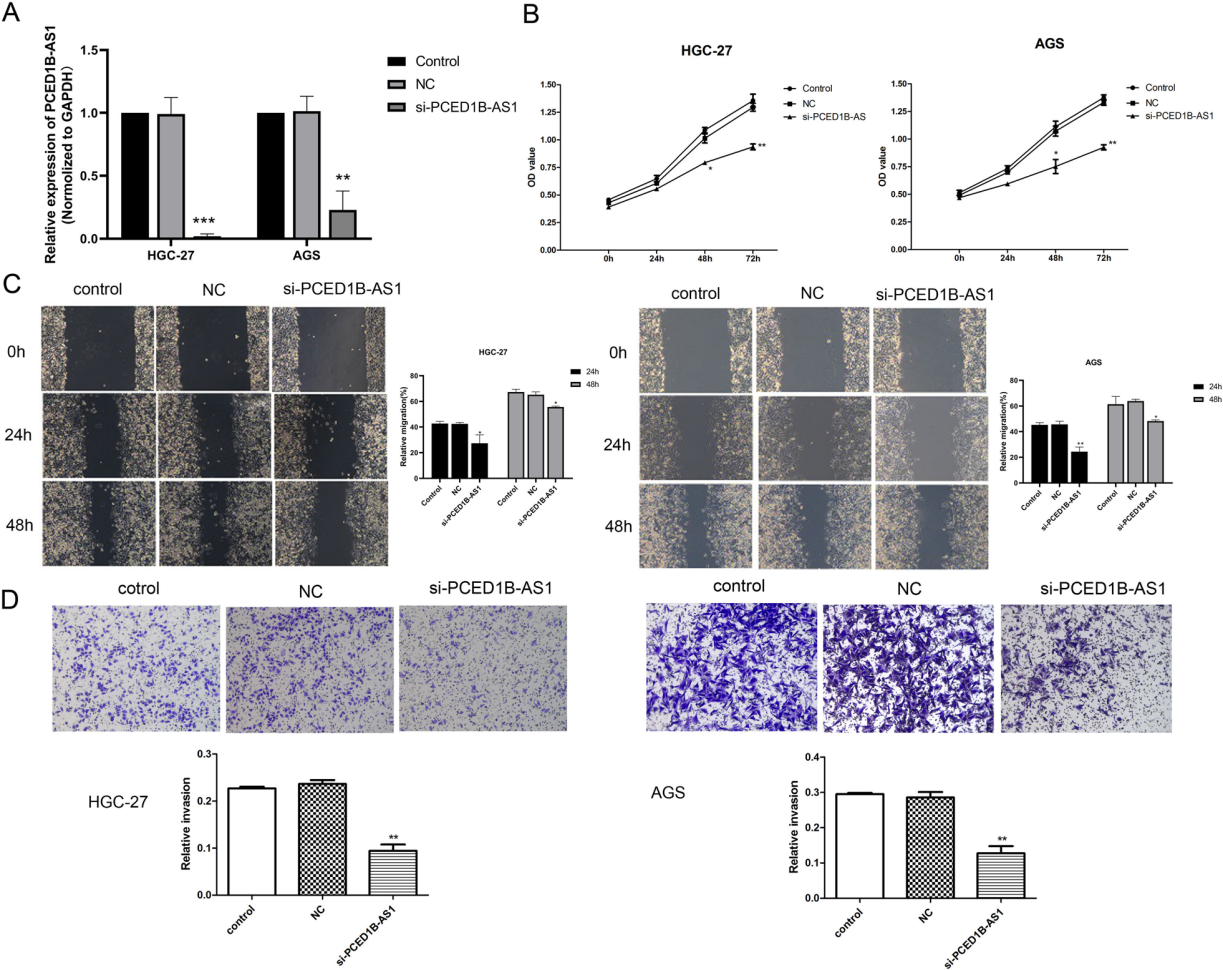
Knockdown PCED1B-AS1 inhibited cell viability, invasion and migration in gastric cancer cell line. **A** RT-*q*PCR analysis was performed to detect the mRNA expression of PCED1B-AS1 in GC cell lines (AGS and HGC-27). **B** CCK-8 kit was used to assess the effect of PCED1B-AS1 on cell viability in AGS and HGC-27. **C** Wound healing assay was used to detect the migration in AGS and HGC-27. **D** Transwell experiment was performed to detect the invasion in AGS and HGC-27. The data were presented as mean ± SEM in three independent experiments. Versus NC group, *P < 0.05, **P < 0.01, n = 3

Next, to further explore the function of PCED1B-AS1 on gastric cancer cells, the function of gastric cancer cells was detected after transfection si-PCED1B-AS1. RT-*q*PCR analysis was performed to detect the mRNA expressions of PCED1B-AS1 in AGS and HGC-27 cells. As shown in Fig. 2A, compared to NC group, the mRNA expression of PCED1B-AS1 in si-PCED1B-AS1 group was reduced significantly. CCK-8 kit results (Fig. 2B) revealed that compared with NC group, the cell viability in si-PCED1B-AS1 group was decreased significantly. And wound healing results (Fig. 2C) showed that compared with NC group, the migration in si-PCED1B-AS1 group was reduced significantly. Transwell experiment results (Fig. 2D) showed that si-PCED1B-AS1 could inhibit the invasive ability. To sum up, we found that knockdown PCED1B-AS1 inhibited cell viability, invasion and migration in gastric cancer cell line.

### Knockdown PCED1B-AS1 inhibited EMT in gastric cancer cell line

**Fig. 3:**
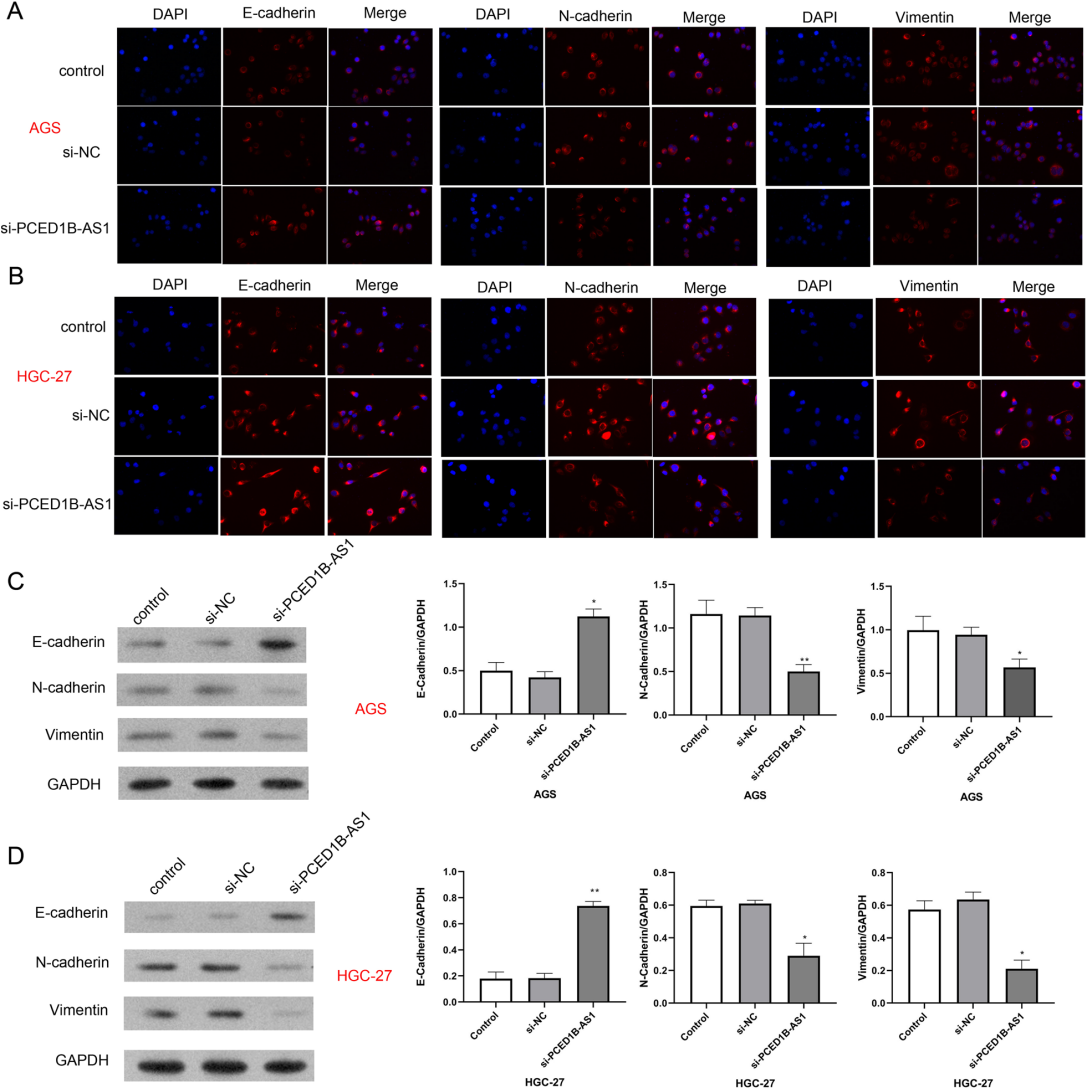
Knockdown PCED1B-AS1 inhibited the EMT process in gastric cancer cell line. Immunofluorescence staining was used to detect the expression of E-cadherin, N-cadherin and Vimentin in AGS cells (**A**) and HGC cells (**B**). The nucleus was labeled with DAPI (blue). Western blotting assay was performed to detect the protein expressions of E-cadherin, N-cadherin and Vimentin in AGS cells (**C**) and HGC-27 cells (**D**). The data were presented as mean ± SEM in three independent experiments. Versus si-NC group, *P < 0.05, **P < 0.01, n = 3

Furthermore, the expression levels of EMT-related proteins (E-cadherin, N-cadherin and Vimentin) were detected by immunofluorescence staining and western blotting assay. The results (Fig. 3A, B) showed that compared with NC group, the positive signal of E-cadherin was significantly increased in si-PCED1B-AS1 group, while the positive signal of N-cadherin and Vimentin was significantly reduced. Western blotting results (Fig. 3C, D) showed that compared with NC group, the protein expressions of E-cadherin were significantly increased in si-PCED1B-AS1 group, the protein expressions of N-cadherin and Vimentin were reduced. Taken together, we confirmed si-PCED1B-AS1 significantly promoted the expression of E-cadherin, inhibited the expression of N-cadherin and Vimentin in AGS and HGC-27 cells.

### PCED1B-AS1 located in the cytoplasm of gastric cancer cells

**Fig. 4:**
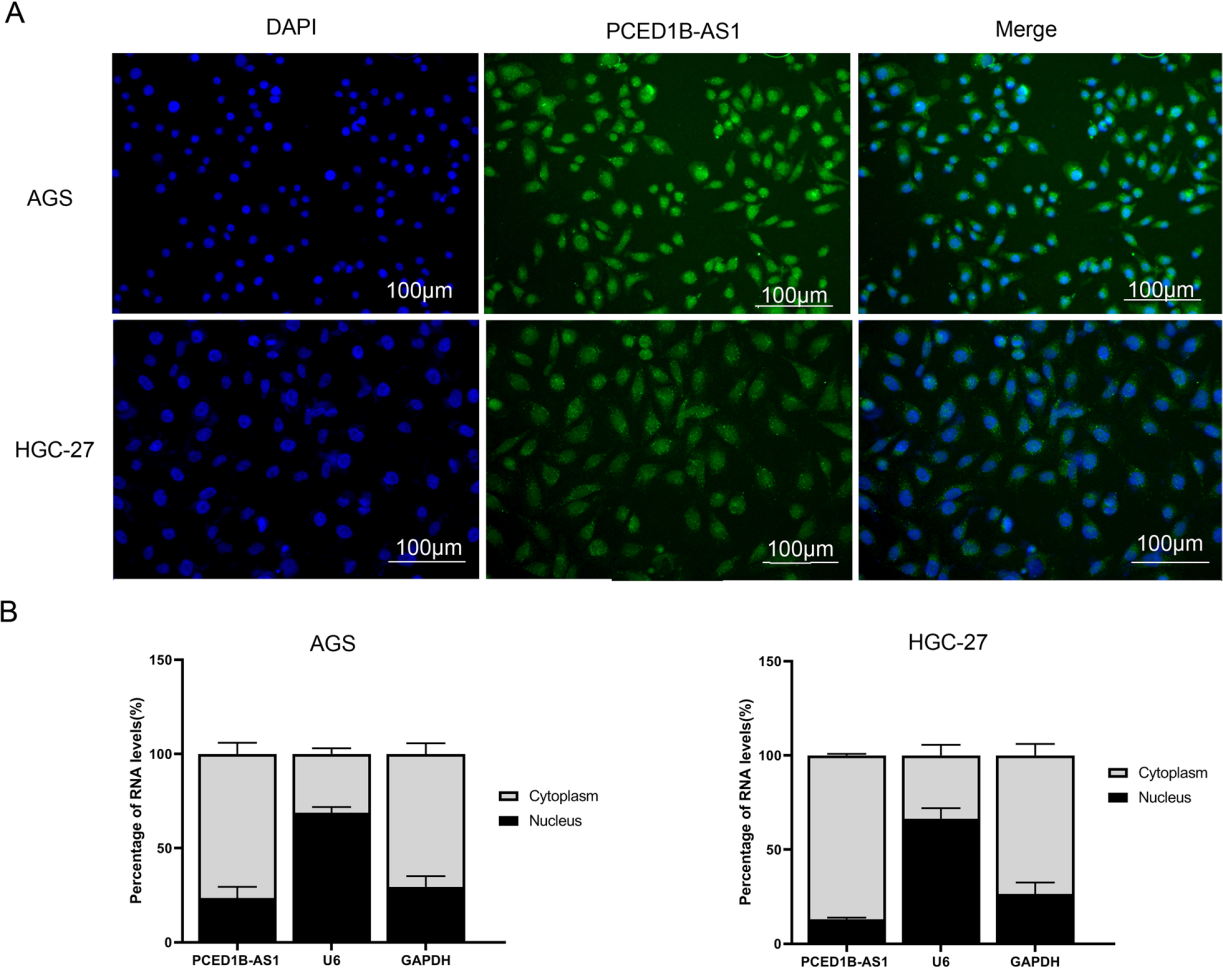
PCED1B-AS1 located in the cytoplasm of gastric cancer cells. **A** Localization of PCED1B-AS1 in gastric cancer cells was detected by fluorescence in situ hybridization (FISH). Nuclei were stained with DAPI. **B** RT-*q*PCR analysis was used to detect the mRNA expression of PCED1B-AS1 in cytoplasm and nucleus. The data were presented as mean ± SEM, n = 3 each group

To further study the mechanism of PCED1B-AS1 in gastric cancer cells, we used fluorescence in situ hybridization (FISH) to study its localization in gastric cancer cells. The results (Fig. 4A) showed that PCED1B-AS1 is localized in the cytoplasm of gastric cancer cells. Then, RT-*q*PCR analysis was performed to detect the mRNA expressions of PCED1B-AS1 in the cytoplasm and nucleus. RT-*q*PCR results (Fig. 4B) showed that compared to the mRNA expressions of PCED1B-AS1 in the nucleus, the mRNA expressions in the cytoplasm were higher. Combined with the above results, we speculated that PCED1B-AS1 is located in the cytoplasm and plays a regulatory role in gastric cancer cells.

### PCED1B-AS1 mediated MAP2K7 to affect the function of gastric cancer cells

**Fig. 5:**
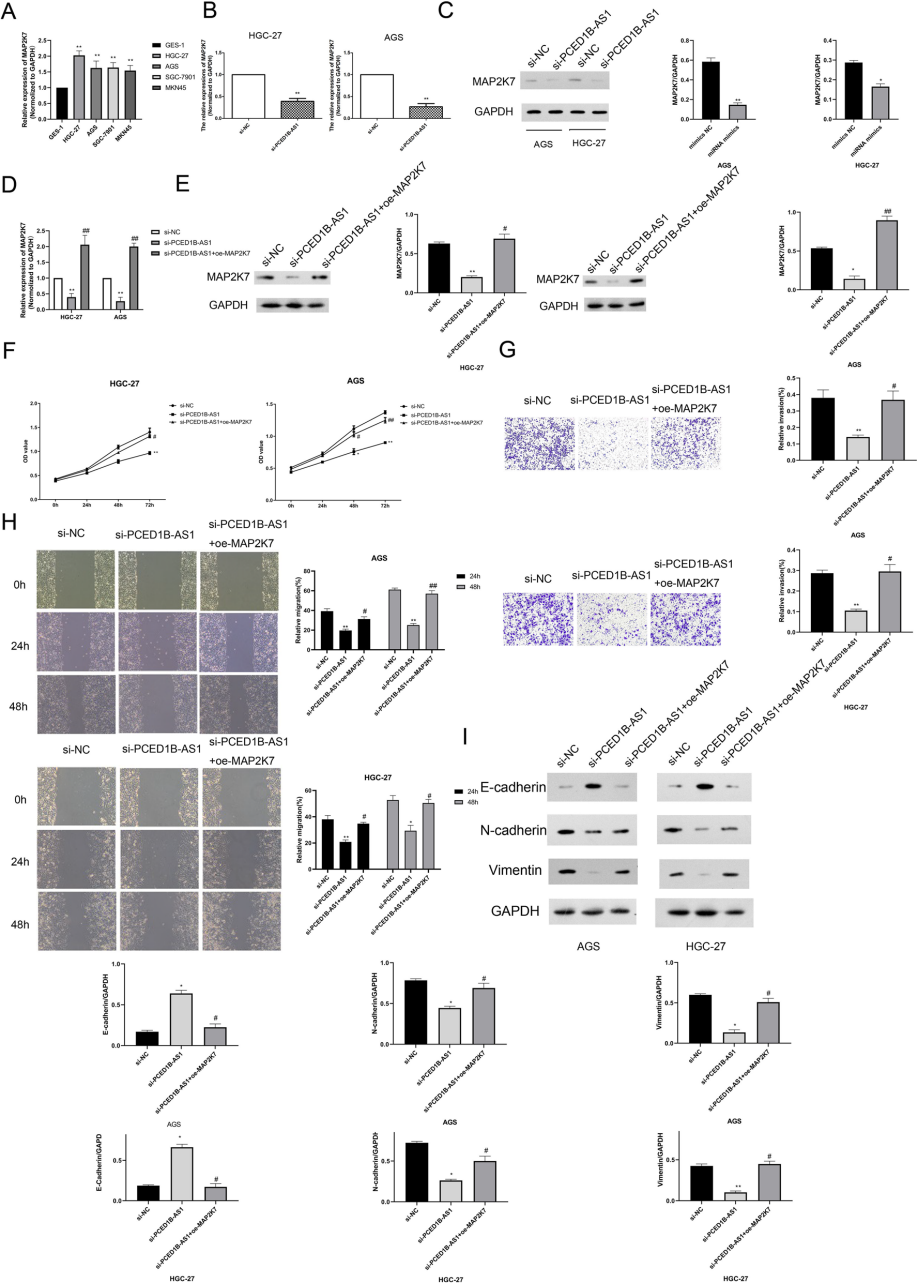
PCED1B-AS1 mediated MAP2K7 to affect the function of gastric cancer cells. **A** RT-*q*PCR analysis was performed to detect the mRNA expressions of MAP2K7 in gastric cancer cells. **B** RT-*q*PCR analysis was performed to detect the mRNA expressions of MAP2K7 in different groups. **C** Western blotting assay was used to detect the protein expressions of MAP2K7 in different groups. **D** After transfection with si-PCED1B-AS1 and oe-MAP2K7, the mRNA expression of MAP2K7 was detected by RT-*q*PCR analysis. **E** The protein expressions of MAP2K7 in different groups were detected by western blotting assay. **F** Cell viability was assessed by CCK-8 kit. **G** Transwell experiment was used to detect the invasion in different groups. **H** Wound healing assay was used to detect the migration in different groups. The expressions of E-cadherin, N-cadherin and Vimentin in different groups were detected by western blotting assay. The data were presented as mean ± SEM, versus si-NC group, *P < 0.05, **P < 0.01; versus si-PCED1B-AS1 group, ^#^*P* < 0.05, ^##^*P* < 0.01, n = 3

Mass spectrometric analysis results (Additional file 2: Table S1) confirmed that lncRNA PCED1B-AS1 had a targeted interaction with MAP2K7. Firstly, RT-*q*PCR analysis was used to detect the mRNA expressions of MAP2K7 in different gastric cancer cells. The results (Fig. 5A) showed that compared with GES-1 cell group, the mRNA expressions of MAP2K7 were significantly up-regulated in gastric cancer cells. After transfection with si-PCED1B-AS1, RT-*q*PCR results (Fig. 5B) showed that compared with si-NC group. The mRNA expression of MAP2K7 was reduced significantly. Western blot results (Fig. 5C) showed that compared with si-NC group, the expressions of si-PCED1B-AS1 in si-PCED1B-AS1 group were reduced significantly. Western blot results were consistent with sequencing results. After transfection with si-PCED1B-AS1 and oe-MAP2K7, RT-*q*PCR analysis was used to detect the mRNA expressions of MAP2K7. The results (Fig. 5D) showed that compared with si-NC group, the expressions of MAP2K7 in si-PCED1B-AS1 group were reduced significantly, while the expressions of MAP2K7 in si-PCED1B-AS1 + oe-MAP2K7 group was increased significantly compared to si-PCED1B-AS1 group. Consistent with RT-*q*PCR results, western blot results (Fig. 5E) also showed the same trend. CCK-8 kits results (Fig. 5F) showed that compared with si-NC group, the cell viability in si-PCED1B-AS1 group was reduced significantly. Compared with si-PCED1B-AS1 I group, the cell viability in si-PCED1B-AS1+oeMAP2K7 group was increased significantly. Transwell experiment results (Fig. 5G) showed that compared with si-NC group, the invasion in si-PCED1B-AS1 group was reduced significantly. The invasion in si-PCED1B-AS1+oeMAP2K7 group was increased significantly compared with si-PCED1B-AS1 group. Wound healing results (Fig. 5H) showed that compared with si-NC group, the migration in si-PCED1B-AS1 group was reduced significantly. Compared with si-PCED1B-AS1 group, the migration in si-PCED1B-AS1+oeMAP2K7 group was increased markedly. Furthermore, western blot was used to detect the E-cadherin, N-cadherin and Vimentin in different groups, and to evaluate the effect on EMT in gastric cancer cells. The results (Fig. 5I) showed that compared with si-NC group, the expressions of E-cadherin in si-PCED1B-AS1 group were increased significantly, the expressions of N-cadherin and Vimentin were reduced significantly. Compared with si-PCED1B-AS1 group, the expression of E-cadherin in si-PCED1B-AS1+oeMAPK7 group was reduced significantly, while the expressions of N-cadherin and Vimentin were increased significantly. We hypothesized that PCED1B-AS1 mediated MAP2K7 affects the function of gastric cancer cells.

### PCED1B-AS1 targeting miR-3681-3p affected gastric cancer cell function

**Fig. 6:**
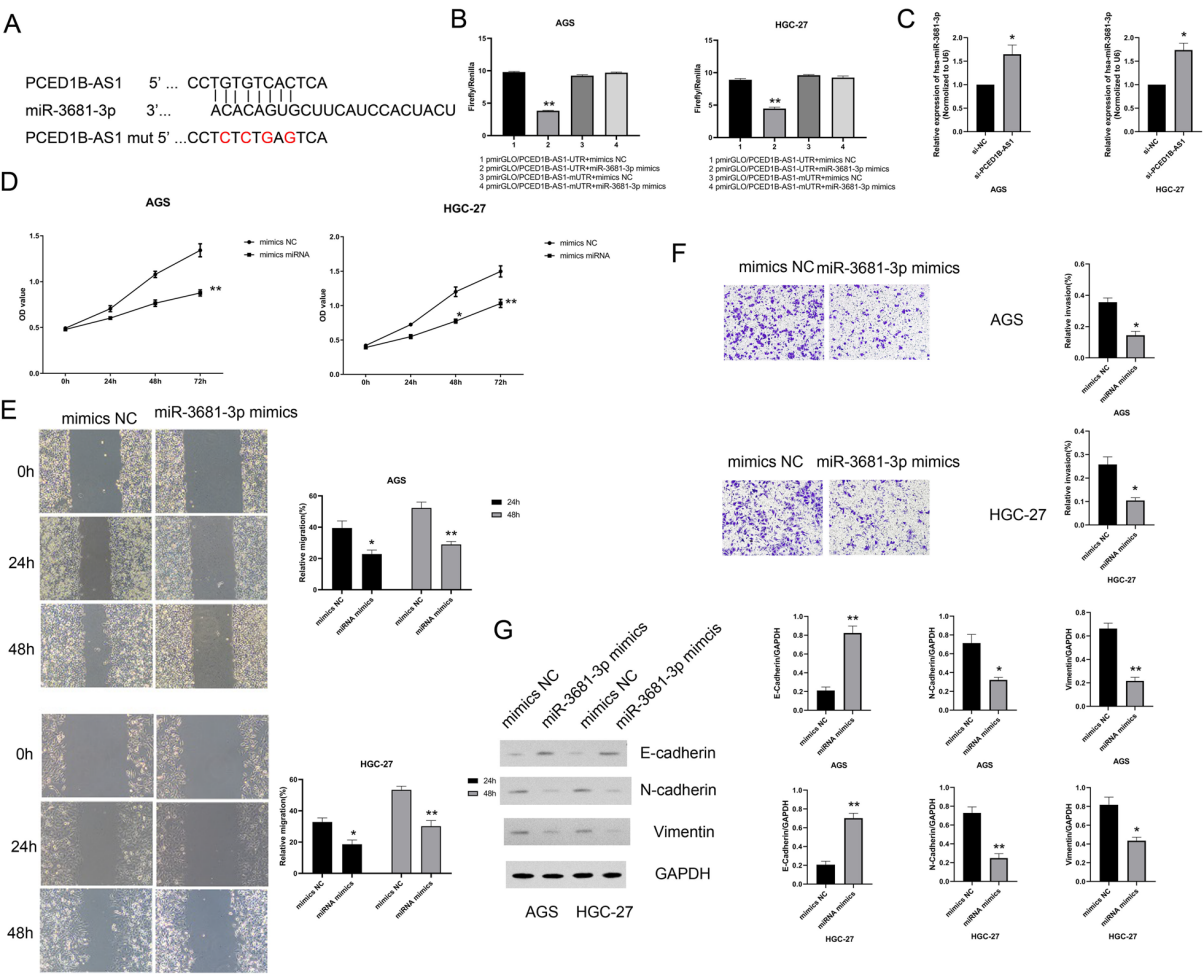
PCED1B-AS1 targeting miR-3681-3p affected gastric cancer cell function. **A** Predicted binding sites and mutant sites of miR-3681-3p on the PCED1B-AS1 transcript. **B** Dual luciferase reporter assay demonstrated that both mimics control or miR-3681-3p mimics were separately transfected into AGS and HGC-27 cells with pmirGLO-PCED1B-AS1 (wide-type) or pmirGLO-PCED1B-AS1 (mutant). **C** RT-*q*PCR analysis was used to detect the expressions of miR-3681-3p in different groups. **D** CCK-8 kit was used to detect the cell viability in different groups. **E** Wound healing assay was performed to detect the migration in different groups. **F** Transwell experiment was used to detect the invasion in different groups. **G** The expressions of E-cadherin, N-cadherin and Vimentin in AGS and HGC-27 cells were detected by western blotting assay. The data were presented as mean ± SEM, versus mimics NC group, *P < 0.05, **P < 0.01

Previous studies have shown that lncRNAs function as molecular spongs of miRNAs; thus, lncRNA PCED1B-AS1 also had sponging activity. Combined with database prediction, we screened miR-3681-3p as downstream targeted regulation of PCEDB-AS1, which severs as a tumor promoter in gastric cancer. RT-*q*PCR analysis results (Additional file 1: Fig. s1) confirmed that compared with GES-1 group, the expressions of miR-3681-3p was significantly down-regulated in gastric cancer cell lines, with significant differences. The dual-luciferase reporter experiment results (Fig. 6A, B) confirmed that compared with mimics NC group, the activity of luciferase in miR-3681-3p mimics group was repressed significantly. It revealed that lncRNA PCED1B-AS1 directed binds to miR-3681-3p in gastric cancer cells. Then, the expressions of miR-3681-3p in different groups were detected by RT-*q*PCR analysis. The results (Fig. 6C) showed that compared with si-NC group, the expression of miR-3681-3p in si-PCED1B-AS1 group was increased significantly. Therefore, we subsequently detected the regulatory role of miR-3681-3p in gastric cancer cells. CCK-8 kit results (Fig. 6D) showed that compared with mimics NC group, the cell viability in miR-3681-3p mimics group was reduced remarkedly. Wound healing results (Fig. 6E) showed that compared with mimics NC group, the migration in miR-3681-3p group was reduced significantly. Transwell experiment results (Fig. 6F) showed that the invasion in miR-3681-3p mimics group was reduced significantly compared with mimics NC group. Besides, western blotting assay was used to detect the protein expressions of E-cadherin, N-cadherin and Vimentin in different groups. The results (Fig. 6G) showed that compared with mimics NC group, the expression of E-cadherin in miR-3681-3p mimics group was increased significantly, the expressions of N-cadherin and Vimentin were reduced significantly. In summary, we confirmed that PCED1B-AS1 targeting miR-3681-3p affected gastric cancer cell function.

### MAP2K7 acts as a sponge of miR-3681-3p in gastric cancer cell

**Fig. 7:**
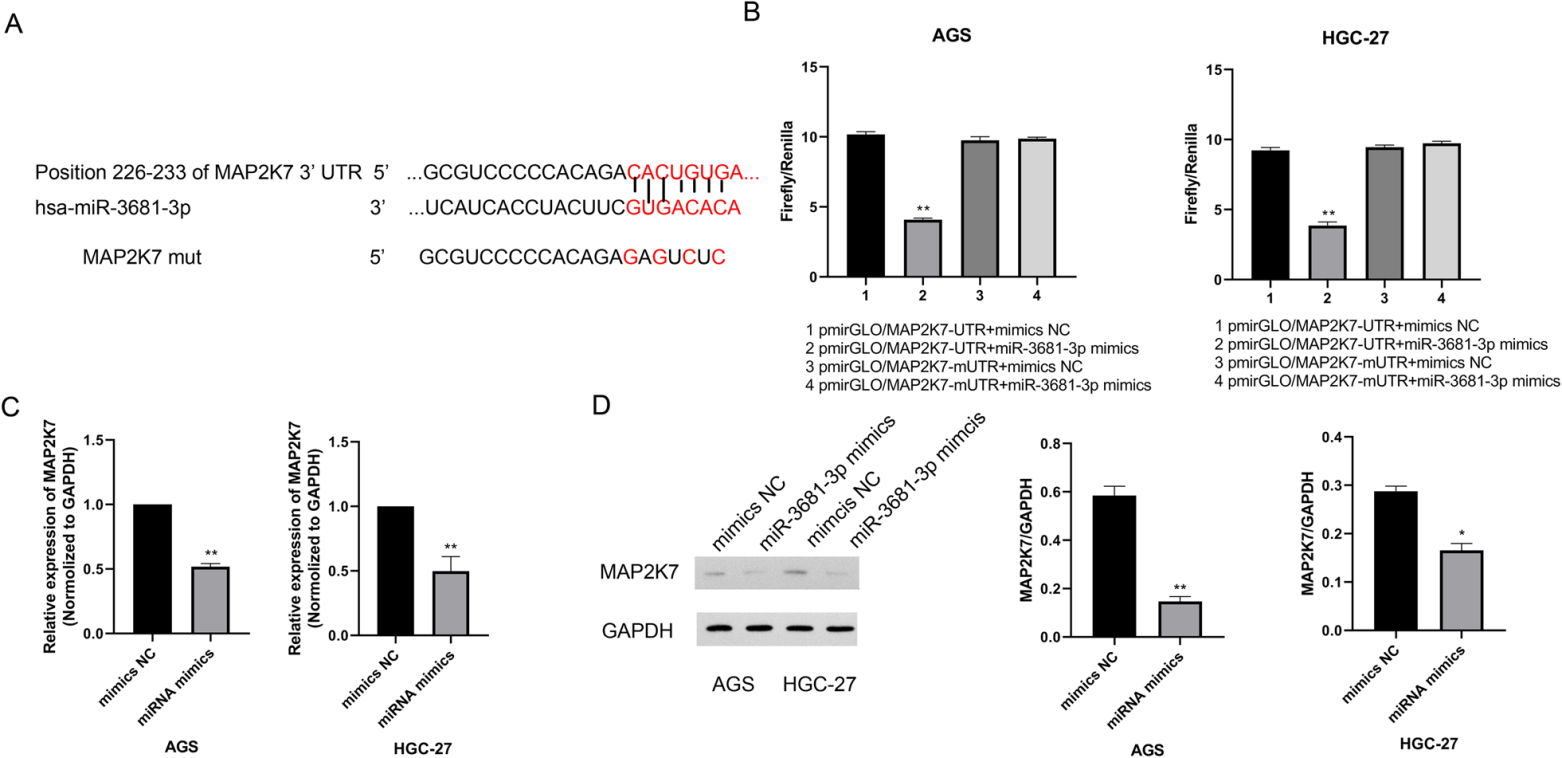
MAP2K7 acts as a spong of miR-3681-3p in gastric cancer cell. **A** Predicted binding sites and mutant sites of miR-3681-3p on the MAP2K7 transcript. **B** Dual luciferase reporter assay demonstrated that both mimics control or miR-3681-3p mimics were separately transfected into AGS and HGC-27 cells with pmirGLO-MAP2K7 (wide-type) or pmirGLO-MAP2K7 (mutant). **C** RT-*q*PCR analysis was used to detect the mRNA expressions of MAP2K7 in AGS and HGC-27 cells. **D** The expressions of MAP2K7 in AGS and HGC-27 cells were detected by western blotting assay. The data were presented as mean ± SEM, versus mimics NC group, *P < 0.05, **P < 0.01, n = 3

Combined with database prediction, we further verified the targeted regulatory relationship between miR-3681-3p and MAP2K7. Luciferase reporter assay results (Fig. 7A, B) confirmed that miR-3681-3p mimics had no effect on the mut-MAP2K7 group in AGS and HGC-27 cells, but significantly reduced the activity in the wt-MAP2K7 group. Additionally, western blotting assay and RT-*q*PCR analysis were used to detect the expressions of MAP2K7 in different groups. RT-*q*PCR results (Fig. 7C) showed that compared with mimics NC group, the expressions of MAP2K7 in miR-3681-3p mimics group were reduced significantly. Similarly, western blot results (Fig. 7D) showed the same trend. RT-qPCR results (Additional file 1: Fig. s1) showed that MAP2K7 expression level was negatively correlated with miR-3681-3p expression in gastric cancer tissues. Taken together, we confirmed that MAP2K7 is a target of miR-3681-3p in gastric cancer cells.

### PCED1B-AS1 mediated miR-3681-3p/MAP2K7 signaling axis to regulate gastric cancer cell function

**Fig. 8:**
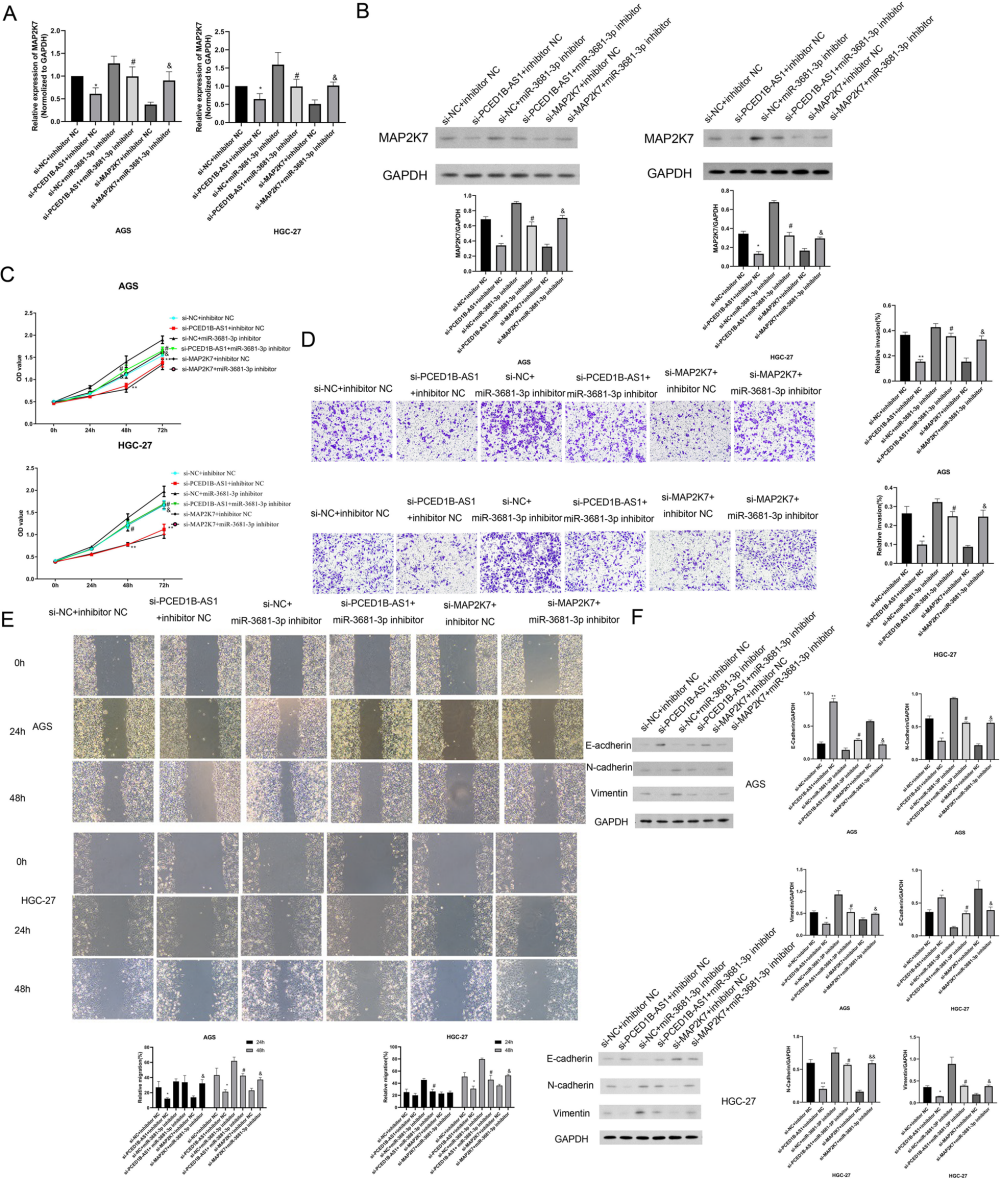
PCED1B-AS1 mediated miR-3681-3p/MAP2K7 signaling axis to regulate gastric cancer cell function. **A** RT-*q*PCR analysis was used to detect the mRNA expressions of MAP2K7 in AGS and HGC-27 cells. **B** Western blotting assay was performed to detect the expressions of MAP2K7 in AGS and HGC-27 cells. **C** The cell viability was assessed by CCK-8 kit. **D** Transwell experiment was performed to detect the invasion in AGS and HGC-27 cells. **E** Wound healing assay was used to detect the migration in AGS and HGC-27 cells. **F** The expressions of E-cadherin, N-cadherin and Vimentin in AGS and HGC-27 cells were detected by western blotting assay. The data were presented as mean ± SEM, versus mimics NC group, *P < 0.05, **P < 0.01; versus si-NC + miR-3681-3p inhibitor group, ^#^*P* < 0.05, ^##^*P* < 0.01; versus si-MAP2K7 + inhibitor NC group, ^&^*P* < 0.05, n = 3

To further explore the molecular mechanism of PCED1B-AS1 in gastric cancer cells, the cell function was studied by using CCK-8 kit, Transwell experiment and wound healing assay, respectively. RT-*q*PCR results (Fig. 8A) showed that compared with si-NC + inhibitor NC group, the expressions of MAP2K7 were reduced significantly in si-PCED-AS1 + inhibitor NC group. While, the expression of MAP2K7 was increased in si-NC + miR-3681-3p inhibitor group compared with si-PCED1B-AS1 group. Compared with si-MAP2K7 + inhibitor NC group, the expression of MAP2K7 was increased significantly in si-MAP2K7 + miR-3681-3p inhibitor group. Similarly, western blot results (Fig. 8B) showed the same trend. The results confirmed that PCED1B-AS1 mediated miR-3681-3p affects the expressions of MAP2K7 in gastric cancer cells(AGS and HGC-27). CCK-8 results (Fig. 8C) showed that compared with si-NC + inhibitor NC group, the cell viability was reduced significantly in si-PCED1B-AS1 + inhibitor NC group. The cell viability was increased in si-NC + miR-3681-3p inhibitor group compared with si-PCED1B-AS1 group. Compared with si-MAP2K7 + inhibitor NC group, the cell viability was increased significantly in si-MAP2K7 + miR-3681-3p inhibitor group. Transwell experiment results (Fig. 8D) showed that compared with si-NC + inhibitor NC group, the invasion was reduced significantly in si-PCED-AS1 + inhibitor NC group. While, the invasion was increased in si-NC + miR-3681-3p inhibitor group compared with si-PCED1B-AS1 group. Compared with si-MAP2K7 + inhibitor NC group, the invasion was increased significantly in si-MAP2K7 + miR-3681-3p inhibitor group. Wound healing results (Fig. 8E) indicated that compared with si-NC + inhibitor NC group, the migration was reduced significantly in si-PCED-AS1 + inhibitor NC group. While, the migration was increased in si-NC + miR-3681-3p inhibitor group compared with si-PCED1B-AS1 group. Compared with si-MAP2K7 + inhibitor NC group, the migration was increased significantly in si-MAP2K7 + miR-3681-3p inhibitor group. Additionally, the expressions of EMT-related proteins were detected by western blot. It (Fig. 8F) showed that compared with si-NC + inhibitor NC group, the expressions of N-cadherin and Vimentin were reduced significantly in si-PCED-AS1 + inhibitor NC group, the expression of E-cadherin was increased significantly. While, the expressions of N-cadherin and Vimentin were increased in si-NC + miR-3681-3p inhibitor group, the expression of E-cadherin was reduced, compared with si-PCED1B-AS1 group. Compared with si-MAP2K7 + inhibitor NC group, the expressions of N-cadherin and Vimentin were increased significantly in si-MAP2K7 + miR-3681-3p inhibitor group, the expression of E-cadherin was reduced. In summary, it suggested that PCED1B-AS1 mediated miR-3681-3p/MAP2K7 signaling axis to regulate gastric cancer cell function.

### Knockdown PCED1B-AS1 inhibited the progression of gastric cancer in mice

**Fig. 9:**
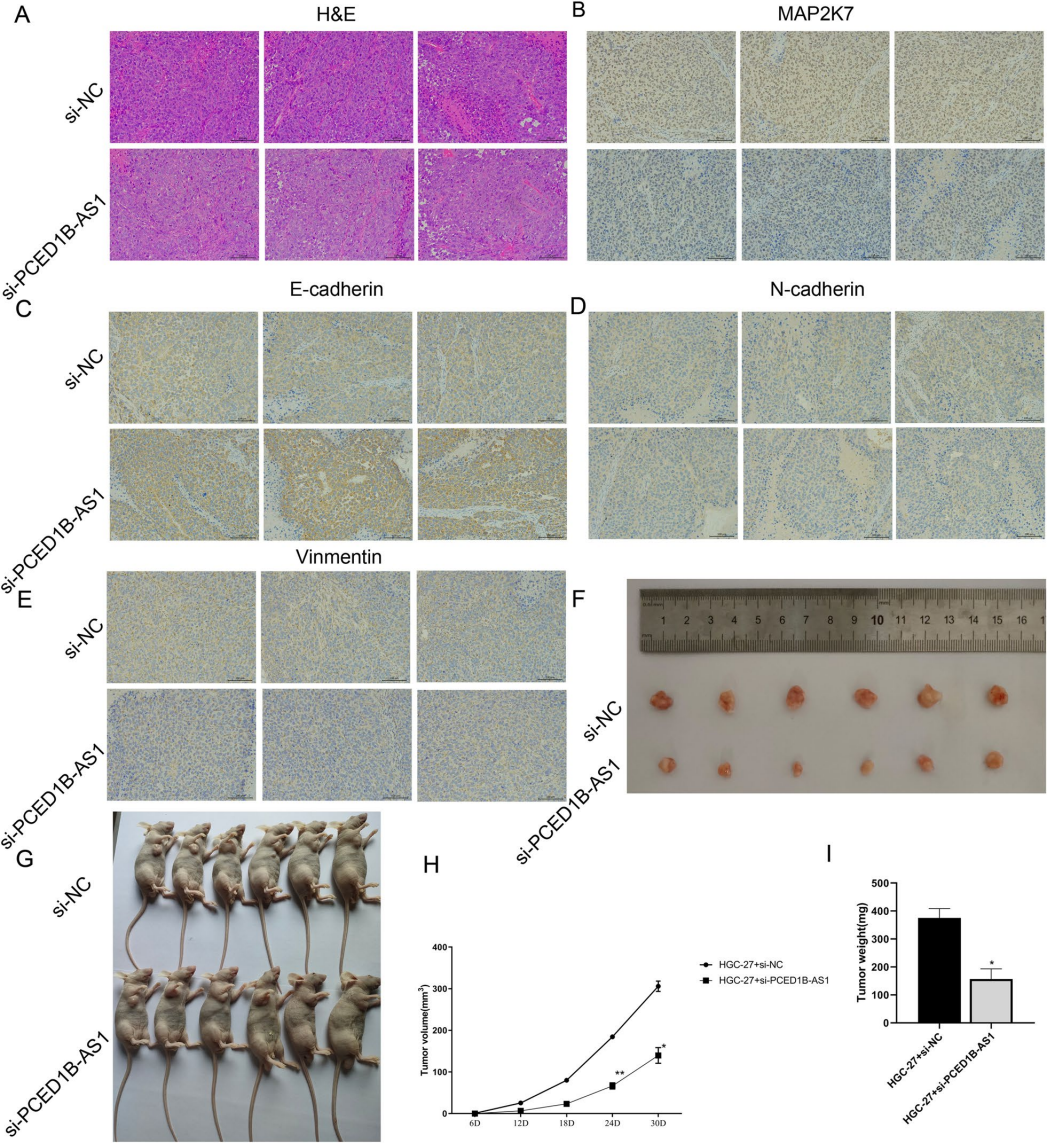
Knockdown PCED1B-AS1 inhibited the progression of gastric cancer in mice. **A** The histopathological changes of gastric cancer were detected by H&E staining. Immunohistochemical staining was used to detect the expression of MAP2K7 (**B**), E-cadherin (**C**), N-cadherin (**D**) and Vimentin (**E**) in gastric cancer tissues. **F** Representative tumor tissue. **G** Representative nude mice are shown. **H** Statistical analysis of tumor volume (**H**) and tumor weight (**I**) from tumors obtained from two groups. The data were presented as mean ± SEM, versus si-NC group, *P < 0.05, **P < 0.01, n = 6

Next, we studied the effect of PCED1B-AS1 on gastric cancer in vivo*.* H&E staining results (Fig. 9A) showed that compared with si-NC group, the necrotic cells that were stained by eosin, and pyknosis was also observed in the tumor tissue in si-PCED1B-AS1 group. Immunohistochemical staining was used to detect the expressions of MAP2K7, E-cadherin, Vimentin and N-cadherin in tissues. It showed that compared with si-NC group, the expressions of MAP2K7 (Fig. 9B), Vimentin (Fig. 9D) and N-cadherin (Fig. E) in si-PCED1B-AS1 were reduced significantly, the expression of E-cadherin (Fig. 9C) was increased. And the tumor tissues were weighed and the volume was measured, respectively. It (Fig. 9F–I) showed that compared with si-NC group, the weight and volume of tumor in si-PCED1B-AS1 group were reduced significantly. Taken together, knockdown PCED1B-AS1 could inhibit the progression of gastric cancer in mice.

## Discussion

As the mortality rate of gastric cancer is a serious threat to the global health system, it has brought great health threat and economic pressure to patients and society [13]. The study of the pathological mechanism of gastric cancer and the potential development of new drugs have attracted extensive attention. In the present study, we found that the expression level of PCED1B-AS1 was abnormally high in gastric cancer cell lines. Furthermore, knockdown of PCED1B-AS1 could significantly inhibit the invasion, proliferation and migration of gastric cancer cells, which may be related to the regulation of miR-3681-3p/MAP2K7 axis.

Previous studies have shown that lncRNAs can play a vital role through sponging miRNAs, affecting the pathological processes in a variety of cancers [14, 15], including proliferation, autophagy [16] and oxidative stress [17]. LncRNA LINC may affect the proliferation, migration and invasion of gastric cancer cells through sponging miR-21 [18]. LncRNA NORAD promotes gastric cancer cell proliferation by regulating miR-608 and affecting FOXO6 protein expression [19]. Ren et al. [20] reported that PCED1B-AS1 accelerated GC progression via adsorbing miR-215-3p and up-regulating CXCR1, indicating that PCED1B-AS1 is a novel therapeutic target for treating GC. Likewise, we further studied the downstream miRNAs of lncRNA PCED1B-AS1 and found specific binding sites between PCED1B-AS1 and miR-3681-3p. Dual-luciferase assay results also confirmed the binding relationship between PCED1B-AS1 and miR-3681-3p. We found that lncRNA PCED1B-AS1 negatively regulated the expression of miR-3681-3p in AGS and HGC-27 cells, affecting cell function. Consistent with previously reported results [20], it suggested that lncRNA PCED1B-AS1 may play a promoting role in the malignant development of gastric cancer.

Previously [17], miR-3681-3p has been reported to be involved in hepatitis B virus infection and may lead to liver cancer. Circ_SIRT1 could mediate miR-3681-3p to affect SIRT1 expression and promote autophagy to alleviate cardiac hypertrophy [21]. Wang et al. [22] found that miR-3681-3p could competitively inhibit the expression of NEK2 and affect the malignant biological behavior of lung adenocarcinoma. Interestingly, this study is the first to demonstrate that miR-3681-3p competitively inhibits MAP2K7 expression and inhibits proliferation, invasion, migration and EMT processes in gastric cancer cell lines.

MAP2K7, also known as MKK7, is a member of the mitogen-activated protein kinase (MAPK) family and is involved in the activation (phosphorylation) of c-Jun N-terminal kinase (JNK) (MAPK), affecting the expression of downstream transcription factors or proliferation-related proteins [23]. Xiao et al. [24] reported that miR-125b targeting MA2K7 regulates MAPK/STAT3 signaling to inhibit osteosarcoma cell proliferation. Tang et al. [25] also suggested that Alpinetin inhibited the proliferation of human liver cancer cells and prevented the malignant development of liver cancer by activating MAP2K7. Histone deacetylase 6 promoted glioblastoma growth through the MAP2K7/JNK/C-Jun signaling pathway [26]. Our findings showed that MAP2K7, as a downstream target of miR-3681-3p and lncRNA PCED1B-AS1, promoted gastric cancer cell proliferation. As the research progresses, we suggested that MAP2K7 overexpression could reverse the effects of pcED1B-AS1 silencing on cell proliferation, invasion and migration. However, miR-3681-3p inhibitors can significantly promote proliferation, invasion, invasion and EMT progression, which is also associated with MAP2K7 regulation.

In conclusion, our study confirmed that silencing lncRNA PCED1B-AS1 directly regulates miR-3681-3p/MAP2K7 signaling axis and inhibited proliferation, invasion and EMT processes of gastric cancer cell lines. More and more reports have confirmed that non-coding RNAs have complex regulatory networks, that is, multiple downstream target molecules, which regulated disease progression in multiple ways [27]. Therefore, the downstream molecule of lncRNA PCED1B-AS1 in the progression of gastric cancer needs to be further studied to clarify its specific marker in gastric cancer. In the future study, we will select more specific target molecules to construct the regulatory network of lncRNA in gastric cancer, so as to provide theoretical support for clinical research.

### Supplementary Information

**Additional file 1: Figure s1**. Correlation between miRNA and MAP2K7 gene expression levels.

**Additional file 2. **Mass spectrometry analysis.

## Data Availability

The datasets during the current study are available from the corresponding author on reasonable request.
