# Supplementary figures and images for: LncRNA PCED1B-AS1 mediates miR-3681-3p/MAP2K7 axis to promote metastasis, invasion and EMT in gastric cancer
Source: Biol Direct. 2024 May 2;19:34. doi: 10.1186/s13062-024-00468-z (PMC11064384; doi:10.1186/s13062-024-00468-z)

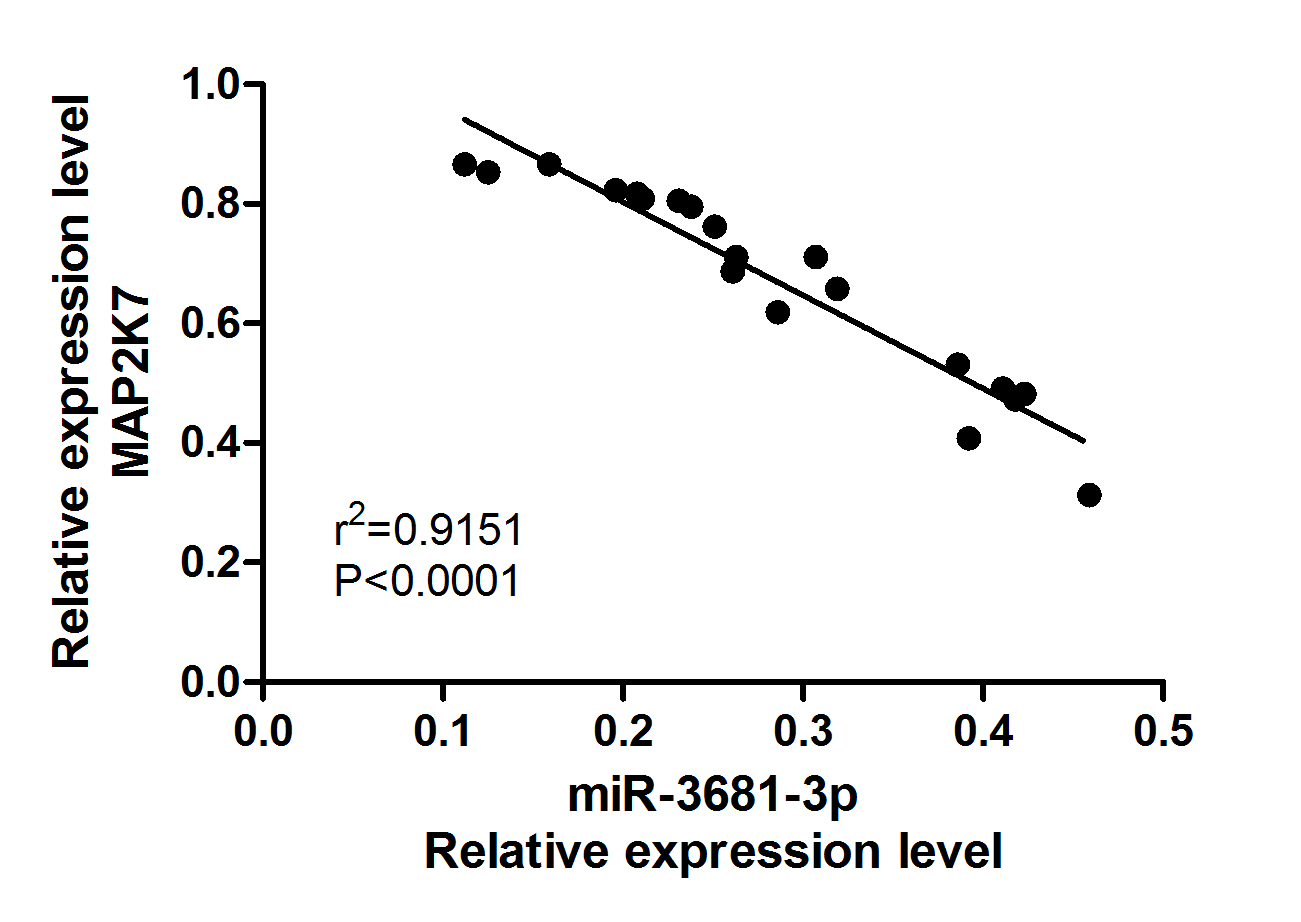

Supplement: Supplementary file 1 — Additional file 1: Figure s1. Correlation between miRNA and MAP2K7 gene expression levels. [file 13062_2024_468_MOESM1_ESM.jpg]
